# Decreased Vancomycin MICs among Methicillin-Resistant *Staphylococcus aureus* Clinical Isolates at a Chinese Tertiary Hospital over a 12-year Period

**DOI:** 10.3389/fmicb.2016.01714

**Published:** 2016-10-27

**Authors:** Chaohui Lu, Yinjuan Guo, Shanshan Wang, Zhengzheng Wang, Lan Chen, Jinnan Lv, Xiuqin Qi, Zengqiang Chen, Lizhong Han, Xueqing Zhang, Liangxing Wang, Fangyou Yu

**Affiliations:** ^1^Department of Respiratory Medicine, The Second Affiliated Hospital of Wenzhou Medical UniversityWenzhou, China; ^2^Department of Laboratory Medicine, The First Affiliated Hospital of Wenzhou Medical UniversityWenzhou, China; ^3^Department of Clinical Microbiology, Ruijin Hospital of Shanghai Jiaotong UniversityShanghai, China; ^4^Department of Respiratory Medicine, The First Affiliated Hospital of Wenzhou Medical UniversityWenzhou, China

**Keywords:** methicillin-resistant *Staphylococcus aureus*, MIC, vancomycin, shift

## Abstract

The increased vancomycin minimum inhibitory concentration values (MICs) for methicillin-resistant *Staphylococcus aureus* (MRSA) isolates are associated with treatment failure and mortality of MRSA infections. In the present study, 553 non-duplicate MRSA isolates from various specimens of patients with infections at a Chinese tertiary hospital from January 2003 to December 2014, were selected randomly for investigating the shift of vancomycin MICs determined by E-test method. The percentages of the MRSA isolates with vancomycin MICs of ≥2.0, 1.5, 1.0, and ≤0.75 mg/L were 16.3% (90/553), 38.5% (213/553), 35.6% (197/553), and 9.9% (55/553), respectively. The highest geometric mean MIC (GM MIC) value (1.648 mg/L) and the lowest GM MIC (0.960 mg/L) were found in the first year (2003) and the last year (2014) over the study period, with significant difference (*p* < 0.05). The GM MICs over the study period fluctuated by year, with the elevated values in 2005, 2011, and 2013 and the decreased values in other years relative to the respective former year. The vancomycin GM MIC (1.307 mg/L) for MRSA isolates from sputum was the highest relative to that for the MRSA isolates from other specimens. By contrast, the vancomycin GM MIC value (1.156 mg/L) for MRSA isolates from pus was the lowest, with similar value to that for the isolates from blood. The vancomycin GM MICs in period I (2003–2005), period II (2006–2008), period III (2009–2011), and period IV (2012–2014) were 1.501, 1.345, 1.177, and 1.139 mg/L, respectively, with the continuous decreased trend. Compared with period I, the vancomycin GM MIC for MRSA isolates in period IV was significantly lower (*p* < 0.01), with a 1.318- fold decrease. The percentages of the isolates with vancomycin MIC ≥2 mg/L in four periods were 25, 15.6, 15.2, and 12%, respectively, with a continuous decrease. While the percentages of the isolates with vancomycin MIC ≤0.75 mg/L in four periods increased from 1.7% in period I to 19.3% in period IV. Taken together, a decreased trend in vancomycin MICs for MRSA isolates from a Chinese tertiary teaching hospital has been found. This pnenomenon was mainly associated with a decrease in the proportion of the MRSA isolates with vancomycin MIC ≥2 mg/L and an increase in the proportion of the MRSA isolates with vancomycin MIC ≤0.75 mg/L.

## Introduction

*Staphylococcus aureus*, particularly MRSA, is an important cause of infections including skin and soft tissue infections, foreign-body infections, pneumonia, septic arthritis, endocarditis, osteomyelitis, sepsis, and bloodstream infections both in hospitals and community settings (David and Daum, 2010). Since the first European isolate of MRSA was detected in 1960s, MRSA infections have become a global concern (Cohen, 2007). In the USA, the mortality caused by MRSA infections had surpassed that caused by acquired immunodeficiency syndrome, hepatitis, or tuberculosis (Klevens et al., 2007). Up to now, the treatment of MRSA infections mainly depends on glycopeptide antibiotics. Vancomycin, a representative of glycopeptide antibiotics, is often used for treating MRSA infections (Levine, 2006). However, the emergence of VISA, hVRSA, and VRSA results in the failure of vancomycin treatment for serious MRSA infections, which is becoming an important public health threat. Although, MRSA isolates with intermediate or resistant to vancomycin remain rare, many reports have described that the extensive use of vancomycin resulted in the increase of vacomycin MICs values, even near to 2 mg/L (Steinkraus et al., 2007; Soriano et al., 2008; Dhand and Sakoulas, 2012). The increased vancomycin MIC values for MRSA isolates were associated with treatment failure and mortality (Martin et al., 2010). Because the high failure rate of vancomycin treatment for MRSA infections is associated with higher vancomycin MICs, the CLSI reduced the vancomycin susceptibility breakpoint for *S. aureus* from MIC value of 4–2 mg/L to improve the predictive susceptibility result (CLSI, 2016). In China, although VRSA is not by far found, the prevalence of hVRSA was more than 10% (Sun et al., 2009) and VISA has been reported (Zhang et al., 2013; Zhu et al., 2015). The elevated vancomycin MICs among MRSA clinical isolates in China should be of concern. The primary objective of this study was to evaluate the shift in vancomycin MICs for MRSA clinical isolates over a 12-year period (2003–2014) at a tertiary teaching hospital in China.

## Materials and Methods

### Ethical Statement

The Ethics Committee of The First Affiliated Hospital of Wenzhou Medical College exempted this study from review because it focussed on bacteria.

### MRSA Clinical Isolates

From January 2003 to December 2014, a total of 553 MRSA isolates (single isolate per patient) from various specimens of patients at the First Affiliated Hospital of Wenzhou Medical University located in Wenzhou, East China, were selected randomly to investigate the shifts in vancomycin MICs. The proportions of MRSA isolates isolated from various specimens were as follows: 67.3% (372/553), sputum; 13.9% (77/553), pus; 6.5%(36/553), exudates; 5.1% (28/553), blood; 4.3% (24/553), catheter and 2.8%(16/553), drainage. The over 12-year study period was divided into four periods including period I (2003–2005), period II (2006–2008), period III (2009–2011), and period IV (2012–2014). Among 553 MRSA isolates, the number of the MRSA isolates in period I (2003–2005), period II (2006–2008), period III (2009–2011), and period IV (2012–2014) were 116, 180, 79 and 158, respectively. The clinical isolates were identified as *S. aureus* using VITEK-2 automatic microbiology analyzer (bioMérieux, Marcy-l’Étoile, France). *S. aureus* ATCC 25923 was used as a control strain for identification of bacteria. Cefoxitin disk diffusion was used for detecting MRSA using MRSA N315 as a positive control strain according to the guidelines provided by CLSI (2016).

### Antimicrobial Susceptibility Testing

Vancomycin MICs for MRSA isolates were determined by E-test method (0.016–256 mg/L; AB bioMérieux, Marcy-l’Étoile, France) in accordance with manufacturer’s guidelines. Interpretive standards for vancomycin susceptibility for MRSA isolates tested were in accordance with the guidelines provided by CLSI (2016). *S. aureus* ATCC 29213 was used as a control train for antimicrobial susceptibility testing. According to the guidelines mentioned above, the *S. aureus* isolates with vancomycin MICs ≤2 mg/L, 2< MICs ≤8 mg/L, and MICs >8 mg/L were regarded as susceptible, intermediate, and resistant to vancomycin, respectively.

### Data Analysis and Statistical Methods

The MIC_50_ (MICs required to inhibit the growth of 50% of organisms), MIC_90_ (MICs required to inhibit the growth of 90% of organisms), MIC range, median MIC and geometric mean MIC (GM MIC) were analyzed. Non-parametric correlation (the Spearman’s ranking correlation coefficient) was used for analyzing the shift in vancomycin MICs over 12-year period. The trend in the proportion of MICs less than or equal to the 2003 median MIC for each individual period was assessed using Mantel–Haenszel X2 test. The statistical analyses were accomplished using SPSS software (SPSS, Chicago, IL, USA). *p*-Value with < 0.05 was considered statistical significance.

## Results

### Prevalence of MRSA Isolates

The prevalence of MRSA isolates in 12-year study period was 58.5% (553/946). The proportions of MRSA isolates among period I (2003–2005), period II (2006–2008), period III (2009–2011), and period IV (2012–2014) were 56.9% (116/204), 68.4% (180/263), 51.0% (79/155), and 48.7% (158/324), respectively.

### Vancomycin MIC-Related Parameters among MRSA Isolates

Of 553 MRSA clinical isolates, 98.0% were susceptible to vancomycin, while 2.0% (11 isolates) with vancomycin MIC = 3 mg/L were intermediate susceptible to vancomycin. The vancomycin MICs for 553 MRSA clinical isolates tested ranged from 0.38 to 3.00 mg/L, with the GM MIC of 1.35 mg/L. The percentages of the MRSA isolates with vancomycin MICs of ≥2.0, 1.5, 1.0, and ≤0.75 mg/L were 16.3% (90/553), 38.5% (213/553), 35.6% (197/553), and 9.9% (55/553), respectively. The highest (1.648 mg/L) and the lowest GM MIC value (0.960 mg/L) were found in the first year (2003) and the last year (2014) during the study period. The vancomycin GM MICs over the study period fluctuated by year, with the increased results in 2005, 2011, and 2013 and the decreased results in other years relative to the respective former year. The vancomycin MIC-related parameters among MRSA isolates from different specimens were showed in **Table 1**. The vancomycin GM MIC value (1.307 mg/L) for MRSA isolates from sputum was the highest relative to that for the MRSA isolates from other specimens. By contrast, the vancomycin GM MIC value (1.156 mg/L) for MRSA isolates from pus was the lowest, with similar value to that for the isolates from blood. The vancomycin MIC_50_ values for MRSA isolates from pus and drainage were 1 mg/L, while those for MRSA isolates from other specimens were 1.5 mg/L. The vancomycin MIC_90_ values for MRSA isolates from blood and drainage were 1.5 mg/L. The isolates from sputum, pus, catheter and exudates had vancomycin MIC_90_ value of 2 mg/L.

**Table 1:** Vancomycin MIC-related parameters of MRSA isolates from different specimens.

Specimens	Geometric mean MIC (mg/L)	MIC_50_ (mg/L)	MIC_90_ (mg/L)	MIC range (mg/L)	MIC ≥2 mg/L (%)	1 ≤ MIC < 2 mg/L (%)	MIC <1 mg/L (%)
Sputum	1.307	1.5	2	0.5–3	16.7	75.7	7.6
Pus	1.156	1	2	0.38–3	17.7	64.6	17.7
Blood	1.188	1.5	1.5	0.38–2	10.7	78.6	10.7
Catheter	1.261	1.5	2	0.38–3	20.0	64.0	16.0
Exudates	1.243	1.5	2	0.5–2	12.8	64.1	23.1
Drainage	1.247	1	1.5	1–2	6.3	93.7	0


### Comparison of Vancomycin MIC-Related Parameters for MRSA Isolates in 4 Study Periods

The vancomycin GM MIC values in period I (2003–2005), period II (2006–2008), period III (2009–2011), and period IV (2012–2014) were 1.501, 1.345, 1.177, and 1.139 mg/L, respectively, with the continuous decreased trend (**Table 2**). Compared with period I, the vancomycin geometric mean MIC value for MRSA isolates in period IV was significantly lower (*p* < 0.01), with a 1.318- fold decrease. The vancomycin MIC_50_ values in period I and period II were 1.5 mg/L, while those in the latter two periods were 1.0 mg/L. The vancomycin MIC_90_ values in four periods all were 2.0 mg/L. The vancomycin MIC for all MRSA isolates in 2003 was ≥1 mg/L, while only 66.7% of the isolates tested with MIC ≥1 mg/L were found in 2014. The percentage of MRSA isolates with vancomycin MIC ≥2 mg/L in 2003 was high to 45.5%, while that was not found in 2014. The percentages of MRSA isolates in four periods with different vancomycin MICs were showed in **Table 3**. The percentages of the MRSA isolates with vancomycin MIC ≥2 mg/L in period I (2003–2005), period II (2006–2008), period III (2009–2011), and period IV (2012–2014) were 25, 15.6, 15.2, and 12%, respectively, with a gradual decrease. While the percentages of the isolates with vancomycin MIC ≤0.75 mg/L in four periods mentioned above increased from 1.7% in period I to 19.3% in period IV (**Table 3**). The percentages of the isolates with vancomycin MIC = 1 mg/L and MIC = 1.5 mg/L in four periods fluctuated. The vancomycin MICs for 75% of the isolates in period I, 84.4% of the isolates in period II, 84.8% of the isolates in period III and 88% of the isolates in period IV were ≤1.5 mg/L. The percentages of MRSA isolates with MICs less than or equal to median MIC (1.5 mg/L) in period I (2003–2005) in four periods were 75, 84.4, 84.8, and 88%, respectively.

**Table 2:** Vancomycin MIC-related parameters in four study periods (mg/L).

Periods	Geometric mean MIC	MIC_50_	MIC_90_	MIC range
I (2003–2005)	1.501	1.5	2.0	0.75–3.0
II (2006–2008)	1.345	1.5	2.0	0.5–3.0
III (2009–2011)	1.177	1	2.0	0.38–3.0
IV (2012–2014)	1.139	1	2.0	0.38–3.0


**Table 3:** Percentages of MRSA isolates with different vancomycin MICs (%).

Periods	MIC ≤0.75 mg/L	MIC = 1 mg/L	MIC = 1.5 mg/L	MIC ≥2 mg/L
I (2003–2005)	1.7	19.8	53.5	25
II (2006–2008)	3.3	33.3	47.8	15.6
III (2009–2011)	12.7	44.3	27.8	15.2
IV (2012–2014)	19.3	35.9	32.8	12


## Discussion

In recent years, a continuing increase in the prevalence of MRSA has been found (Kock et al., 2010). Up to now, vancomycin is still a standard first-line antibiotic for MRSA infections, but its MIC creep for MRSA isolates is becoming a major concern. Soriano et al. (2008) suggested that mortality of MRSA bacteremia was significantly higher when vancomycin was empirically used for treating infection caused by the strains with a high vancomycin MIC (>1 mg/L). Lodise et al. (2008) reported that the median hospital length of stay was longer for the patients infected by MRSA isolates with vancomycin MIC ≥1.5 mg/L than that with vancomycin MIC <1.5 mg/L (21 days versus 10.5 days, respectively). In the present study, the percentage of MRSA isolates with vancomycin MIC ≥1.5 mg/L was 55.6% and the geometric mean MICs ranged from 0.96 to 1.648 mg/L over 12 years, which was higher than a previous report from China among which vancomycin geometric mean MICs for 1411 MRSA isolates from six hospitals between 2006 and 2011 were from 0.906 to 1.040 mg/L (Zhuo et al., 2013). Vancomycin MIC values determined by E-test method were higher than those determined by CLSI-recommended broth dilution, MicroScan and Sensititre broth microdilution assays (Prakash et al., 2008; Pitz et al., 2011). 11 MRSA isolates with vancomycin MIC = 3 mg/L determined by E-test method in the present study were re-determined to be vancomycin MIC = 3 mg/L determined by CLSI-recommended broth micro-dilution. The reasons why the vancomycin MIC values for MRSA isolates in the present study were relatively higher may be associated with the application of E-test method and the majority of MRSA isolates (67.3%, 372/553) from sputum with higher vancomycin geometric mean MIC value relative to those from other specimens. However, relatively higher vancomycin MIC values for MRSA isolates found in the present study indicated the potential for treatment failure of vancomycin for MRSA infections and should be of concern.

Steinkraus et al. (2007) reported that the vancomycin MICs for MRSA clinical isolates increased in from 2001 to 2005 at a tertiary care institution in the USA. Increased vancomycin MICs for *S. aureus* clinical isolates from 2000 to 2004 were found in a American hospital (Wang et al., 2006). Previous studies reported that a tendency toward decreased susceptibility to vancomycin in MRSA isolates has reemerged in China (Zhuo et al., 2013; Chang et al., 2015). However, many studies did not find this increased trend (Sancak et al., 2013; Goldman et al., 2014). No upward creep in vancomycin MICs for MRSA isolates was found in the UK and Ireland from 2001 to 2007 (Reynolds et al., 2012). A report from Turkey also did not detect a increase in vancomycin MICs among MRSA blood isolates over an 11-year period (Sancak et al., 2013). The SENTRY Antimicrobial Surveillance Program reported that there was no evidence of increased vancomycin resistance during 1998–2003 (Jones, 2006). Musta et al. (2009) demonstrated that the vancomycin MIC distribution was stable over an 11-year span from 1996 to 2006. Alos et al. (2008) reported that no vancomycin MIC creep from 2002 to 2006 was found in a setting with a low usage of vancomycin. Interestingly, in the present study, we observed a decreased trend of vancomycin MICs for MRSA isolates across 12 years (2003–2014). Our results showed that although vancomycin geometric mean MICs for MRSA isolates fluctuated by year, it decreased continuously per 3 years. We also applied frequency distributions of the MRSA isolates with different MIC values and the percentages of MRSA isolates with MICs less than or equal to 2003–2005 median MIC (1.5 mg/L) to demonstrate a decrease in vancomycin MICs during the study period. Although, the increased vancomycin MICs may be associated with the application of E-test method, these results were suitable for observing the shift in vancomycin MICs for MRSA isolates for a long period. The decreased vancomycin MICs in the present study were primarily associated with a decrease in the proportion of the MRSA isolates with MIC ≥2 mg/L and an increase in the proportion of the isolates with MIC ≤0.75 mg/L. Conflicting findings in vancomycin MIC shift may be due to differences in the study periods, geographic locations, use of new antibiotics, vancomycin usage densities, number of enrolled cases, and MIC assays used. Kehrmann et al. (2011) investigated bloodstream MRSA isolates at several hospitals in two German cities from 2004 to 2009 and found that the creep phenomenon varied by region. Unfortunately, we did not acquire the accurate usage density of vancomycin for MRSA infections during study period, which enable us not to know the association between usage density of vancomycin and decreased vancomycin MIC. We speculate that the pnenomenon found in the present study was associated with enforced administration of reasonable antimicrobials usage by Chinese government and increased samples for bacteria culture. The limitations of the present study included vancomycin MICs determined by E-test method rather than the broth microdilution method and unknown molecular lineages and a selection bias of MRSA isolates.

Taken together, a decreased trend in vancomycin MICs for MRSA isolates from a Chinese tertiary teaching hospital has been found. This pnenomenon was primarily associated with a decrease in the percentage of the MRSA isolates with MIC ≥2 mg/L and an increase in the percentage of the MRSA isolates with MIC ≤0.75 mg/L. As fluctuation of vancomycin MICs occurred in some years, a decreased trend in vancomycin MICs for MRSA isolates found in the present study should be further confirmed by continuous investigation in subsequent years.

## Author Contributions

CL, YG, SW, ZW, JL, and XQ performed the laboratory measurements. FY and LW made substantial contributions to conception and design. LH, LW, and FY revised the manuscript critically for important intellectual content. ZC and XZ participated in experimental design and data analysis. FY drafted the manuscript. All authors read and approved the final manuscript.

## Conflict of Interest Statement

The authors declare that the research was conducted in the absence of any commercial or financial relationships that could be construed as a potential conflict of interest.

## Abbreviations

CLSI: Clinical and Laboratory Standards Institute
hVRSA: hetero-resistant *S. aureus;*
MIC: vancomycin minimum inhibitory concentration
MRSA: methicillin-resistant *Staphylococcus aureus;*
VISA: vancomycin-intermediate *S. aureus;*
VRSA: vancomycin-resistant *S. aureus*
